# A Putative Efflux Transporter of the ABC Family, YbhFSR, in *Escherichia coli* Functions in Tetracycline Efflux and Na^+^(Li^+^)/H^+^ Transport

**DOI:** 10.3389/fmicb.2020.00556

**Published:** 2020-04-23

**Authors:** Zhenyue Feng, Defu Liu, Lizi Wang, Yanhong Wang, Zhongjing Zang, Zhenhua Liu, Baifen Song, Liwei Gu, Zhaowei Fan, Siyu Yang, Jing Chen, Yudong Cui

**Affiliations:** College of Life Science and Technology, Heilongjiang Bayi Agricultural University, Daqing, China

**Keywords:** ABC transporters, YbhFSR, YbhF, tetracyclines, single-drug efflux pump, Na^+^/H^+^ transporter

## Abstract

ATP-binding cassette transporters are ubiquitous in almost all organisms. The *Escherichia coli* genome is predicted to encode 69 ABC transporters. Eleven of the ABC transporters are presumed to be exporters, of which seven are possible drug export transporters. There has been minimal research on the function of YbhFSR, which is one of the putative drug resistance exporters. In this study, the *ybhF* gene of this transporter was characterized. Overexpression and knockout strains of *ybhF* were constructed. The ATPase activity of YbhF was studied using the malachite green assay, and the efflux abilities of knockout strains were demonstrated by using ethidium bromide (EB) as a substrate. The substrates of YbhFSR efflux, examined with the minimum inhibitory concentration (MIC), were determined to be tetracycline, oxytetracycline, chlortetracycline, doxycycline, EB, and Hoechst33342. Furthermore, tetracycline and EB efflux and accumulation experiments confirmed that the substrates of YbhFSR were tetracyclines and EB. The MIC assay and the fluorescence test results showed that tetracyclines are likely to be the major antibiotic substrate of YbhFSR. The existence of the signature NatA motif suggested that YbhFSR may also function as a Na^+^/H^+^ transporter. Overexpression of YbhF in *E. coli* KNabc lacking crucial Na^+^/H^+^ transporters conferred tolerance to NaCl, LiCl, and an alkaline pH. Together, the results showed that YbhFSR exhibited dual functions as a drug efflux pump and a Na^+^ (Li^+^)/H^+^ antiporter.

## Introduction

The presence of bacterial drug efflux transporters is a problem for treatment of bacterial infections because efflux pumps can transport drugs or other substrates outside of the cells and thereby reduce the concentration of drugs (Orelle et al., 2019). Most multidrug efflux pumps of eukaryotes belong to the ATP-binding cassette (ABC) family of transporters, while in prokaryotes, five superfamilies of multi-drug efflux pumps can cause antibiotic resistance (Li et al., 2015). Several of these efflux pumps, while having low levels of resistance, are often the first step in resistance, eventually leading to higher levels of resistance by acquiring chromosomal mutations that target antibiotics (Lubelski et al., 2007; Schmalstieg et al., 2012; Chitsaz and Brown, 2017; El Meouche and Dunlop, 2018; Frimodt-Møller et al., 2018). ABC superfamily drug exporters comprise one of these efflux pumps (Huda et al., 2003).

ATP-binding cassette proteins, an ancient and large family, are widely distributed in almost all species from bacteria to yeast, nematodes, fruit flies, plants, and mammals (Davidson et al., 2008; Moussatova et al., 2008; Eitinger et al., 2011; Teichmann et al., 2017). The ABC transporters have a similar topology, with two nucleotide-binding domains (NBDs) and two transmembrane domains (TMDs) (Orelle et al., 2019). The NBD is located in the cytoplasmic membrane and uses the energy released by ATP hydrolysis to extrude pump substrates through the membrane (Dean et al., 2001). The NBDs are highly conserved and contain Walker A, Walker B, and signature motif (Moussatova et al., 2008). The TMDs recognize and mediate the passage of substrates across the cell membrane and usually form a substrate translocation pathway. The TMD is less conservative. The TMDs of most exporters contain six hydrophobic transmembrane spans (Linton and Higgins, 2007; Jones et al., 2009). TMDs and NBDs can be encoded by different genes (usually prokaryotes), or one TMD and one NBD domain are fused into a protein as so-called “half-size” (TMD-NBD or NBD–TMD) or two TMDs and two NBDs are fused into a big protein as so-called “full-size” (TMD-NBD-TMD-NBD or NBD-TMD-NBD-TMD) transporters (Theodoulou and Kerr, 2015).

ATP-binding cassette proteins transport a wide variety of substrates, including peptides, phospholipids, heavy metal chelates, polysaccharides, antibiotics, steroids, amino acids, proteins, and even inorganic ions (Higgins, 1992; Beis, 2015). ABC drug efflux pumps include single drug resistance (SDR) efflux pumps and multidrug resistance (MDR) efflux pumps (Lubelski et al., 2007). In bacteria, some MDR proteins belonging to the ABC transporter family have been studied, such as MsbA (*Escherichia coli*) (Schultz et al., 2011), Sav1866 (*Staphylococcus aureus*) (Dawson and Locher, 2007; Velamakanni et al., 2008; Oliveira et al., 2011), and LmrA (*Lactococcus lactis*) (Pleban et al., 2004; van den Berg Van Saparoea et al., 2005; Venter et al., 2008). Several ABC SDR external pumps for macrolides and doxorubicin have been studied in Gram-positive bacteria, whereas only a few functional ABC SDR transporters are known to exist in Gram-negative bacteria. The only single-drug efflux pump reported in Gram-negative bacteria is MacAB from *E. coli* (Kobayashi et al., 2001; Lin et al., 2009).

The genomic DNA sequences of many microorganisms, including *E. coli*, have been sequenced (Blattner et al., 1997; Hayashi et al., 2006). From these genomic data, all encoded ABC proteins have been analyzed. There are 79 proteins belonging to the ABC family, thus making it the largest protein family in *E. coli* (Ames et al., 1992; Rees et al., 2009). These ABC proteins constitute 69 independent functional systems, and 11 of them are presumed to be exporters (Moussatova et al., 2008), of which seven are possible drug export transporters, i.e., mdlAB (Foo et al., 2014), YbjYZ, YddA, YojHI, YbhFSR, MacB, and MsbA (Nishino and Yamaguchi, 2001). Two of them, MsbA and MacB, have been confirmed as drug export transporters, and YbhFSR is one of the putative drug resistance exporters. Sequence analysis suggested that *ybhF*, *ybhS*, and *ybhR* encode the subunits of an ABC transporter complex. YbhF has two NBDs, and is the predicted ATP-binding component, whereas YbhS and YbhR are predicted membrane components.

In 2016, Yuki Yamanaka et al. screened the genomic Exponential Enrichment System Evolution of Ligands for the identification of binding sites for the unidentified tetracycline transcription factor, YbiH, in the *E. coli* genome. The binding site was the putative drug efflux pump, YbhFSR, of the ABC family, and the *ybhF* gene of the nucleotide binding domain in the operon and the *ybhG* gene belonging to the membrane fusion protein (MFP) family in the same operon were further knocked out. The growth of the control strain and the knockout strain showed that the addition of cefoperazone affected the growth of the *ybhF* knockout strain, and the addition of chloramphenicol affected the growth of the *ybhG* knockout strain. Although this was the first report of this transporter, the present study was mainly aimed at a study of the transcriptional regulator, YhiH, in the operon where YbhFSR is located. There have been no additional functional studies on the YbhFSR transporter or the *ybhF* gene, so the present study characterized the *ybhF* gene in the YbhFSR transporter. The *ybhF* gene belongs to the ATP-binding domain and transfers the substrate by energy released from ATP hydrolysis. If the gene is deleted, the transporter has no energy source and cannot complete transfer of the substrates.

Amino acids 334–574 of the YbhF protein contain a NatA domain, which is involved in the transport of Na^+^, so we studied the transport function of Na^+^ by using the Na^+^ transfer-deficient strain (KNabc) of *E. coli* (Nozaki et al., 1996).

## Materials and Methods

### Bacterial Strains and Plasmids

Bacterial strains and plasmids used in this study are described in Table 1. The strains were cultured in Luria-Bertani (LB) liquid medium at 37°C. *E. coli*/pET-28a and *E. coli*/pET28a-*ybhF* were maintained in LB liquid medium, containing 100 μg/ml added kanamycin. A drug-sensitive strain was constructed by knocking out the *acrB* gene in *E. coli* K-12, and then the *ybhF* gene was knocked out in WT *E. coli* and the *E. coli*△*acrB*. PCR amplification used pKD3 or pKD4 plasmids as templates. Then, target fragment and electrocompetent cells with pKD46 plasmid were added to a chamber and subjected to electric shock with a Bio-Rad electroporation system (Bio-Rad, Hercules, CA, United States). Positive clones on the plate were selected for PCR verification (Supplementary Figure S1 and Supplementary Table S1). The assays were carried out according to the protocol described by Datsenko and Wanner (2000).

**TABLE 1:** Bacterial strains or plasmid.

Plasmids	Characteristic(s) ^a^	Reference or source
pET28a	Overexpression vector	Saved in our laboratory
pMD18-T	Cloning vector	Purchased from TaKaRa
pKD46	bla (Ap ^r^), a helper plasmid for expression of Redλ recombinase	Donated by Guoqiang Zhu
pKD3	Template plasmid for a chloramphenicol resistance gene	Donated by Guoqiang Zhu
pKD4	bla (Ap ^r^), a plasmid carrying FRT-flanked adjacent kan (Km ^r^) gene	Donated by Guoqiang Zhu
pCP20	bla (Ap ^r^) cat (Cm ^r^), a plasmid for expression of FLP recombinase	Donated by Guoqiang Zhu
**Bacterial strains**		
*E. coli* K-12	Wild type	Haerbin Veterinary Research Institute
*E. coli* DH5α *E. coli* BL21	Δ(*arg*F-*lac*169)80 *dlac*Z58(M15)*gln*V44(AS)^–^ *rfb*D1*gyr*A96 recA1 F^–^ *ompT gal dcm lon hsdS_*B*_*(rB^–^mB^–^) λDE3	Purchased from Takara Saved in our laboratory
*E. coli*/pET28a	pET expression vector	This work
*E. coli*/pET28a-*ybhF*	pET28a-*ybhF*, Kan^r^	This work
*E. coli*Δ*ybhF E. coli*Δ*ybhS E. coli*Δ*ybhR*	*E. coli*Δ*ybhF*:Cl ^r^ *E. coli*Δ*ybhS*:Cl ^r^ *E. coli*Δ*ybhR*:Cl ^r^	This work This work This work
*E. coli*Δ*acrB*	*E. coli* Δ*acrB*: Ap ^r^	This work
*E. coli*Δ*acrB*Δ*ybhF*	*E. coli* Δ*acrB*Δ*ybhF*:Cl ^r^	This work

*Escherichia coli* KNabc without three Na^+^/H^+^ transporters was grown overnight at 37°C in LBK medium until the OD_600_ reached 1.0. *E. coli* KNabc and its transformants were cultured in LBK medium at a specified concentration with the addition of NaCl or LiCl, or at a specified pH, and then their growth was determined. The growth assay was conducted according to the protocol described by previous reports (Meng et al., 2017; Wang et al., 2017; Abdel-Motaal et al., 2018).

### Antibiotics and Chemicals

Tetracycline, oxytetracycline, chlortetracycline, doxycycline, ethidium bromide (EB), Hoechst33342 stain, cefoperazone, cefazolin, streptomycin, ampicillin, roxithromycin, chloramphenicol, rifampicin, norfloxacin, deoxycholate, sodium cholate, ofloxacin, doxorubicin, daunorubicin, acridine flavin, and quinine were purchased from Coolaber (Beijing, China), and *ortho*-vanadate was purchased from Sigma-Aldrich (St. Louis, MO, United States). Carbonylcyanide m-chlorophenylhydrazone (CCCP) and reserpine were obtained from MedChemexpress (Monmouth Junction, NJ, United States). Drug-sensitive paper was purchased from Hangzhou Microbial Preparation (Hangzhou, China).

### Antibodies

His-tagged mouse antibodies and goat anti-mouse IgG-horseradish peroxidase antibody were obtained from Nachuan (Beijing, China), and goat anti-mouse IgG/gold was purchased from Bioss (Woburn, MA, United States). Monoclonal antibody to YbhF was prepared by our laboratory. Preparation of monoclonal antibody of YbhF: BALB/c mice were immunized with purified YbhF protein, and spleen cells of the immunized mice were obtained and fused with SP2/0 myeloma cells. One monoclonal hybridoma cell line secreting antibodies were screened and identified. Positive hybridoma cells were injected into mice to obtain ascites, and the ascites was purified using a purification column to obtain monoclonal antibodies.

### Bioinformatics Analysis of YbhFSR

We obtained FASTA format sequences from the National Center for Biotechnology Information (NCBI) for all proteins. ClustalX software^1^ was used to perform multiple sequence alignments of the YbhF and other proteins. The genetic distances were calculated using Mega 7.0 software^2^ and the phylogenetic tree was constructed with Mega 7.0 software by using the neighbor-joining (NJ) method, with 1000 replications. The homology alignment of YbhF with other ATP-binding component of the ABC family was conducted to identify conserved motifs using DNAStar software^3^. The basic physicochemical properties of the protein were predicted using Protparam software^4^. The TMD of YbhFSR was predicted using TMHMM2.0 software^5^. Using EMBL online software^6^, the homology alignment of amino acid sequence 334–574 of the YbhF protein and RB6469 (a sodium ABC transporter ATP-binding protein from *Rhodopirellula baltica* SH 1, Gene ID: 1794229) was done. The result shows that the amino acid sequence identity of these two proteins was 31.3% and the similarity was 55.2% (Supplementary Figure S3).

### Expression and Purification of YbhF

The sequence of YbhF was obtained from the NCBI. We designed specific primers for PCR amplification (*ybhF*-F and *ybhF*-R, Supplementary Table S1) and restriction enzyme sites, and protected bases were located on both sides of the primers. The amplified fragment was cloned into pET-28a carrier to construct the *E. coli* BL21/pET-28a and *E. coli*/pET28a-*ybhF* recombinant plasmids of *E. coli* (Supplementary Figure S2). Double enzyme cutting and sequencing were then performed. These constructed strains were grown in LB liquid medium with 50 μg/ml kanamycin, when the OD_600_ reached 0.5–0.6, then protein expression was induced by the addition of 1 mM isopropyl beta-D-thiogalactopyranoside (IPTG) at 37°C, and the cells were incubated at 180 rpm for 4 h. The solubilized YbhF protein with His_6_-tag was purified from the supernatant flows with nickel–nitrilotriacetic acid resin and performed as previously described (Diao et al., 2011). Protein expression and purification were then analyzed using SDS-PAGE and Western blotting.

### ATPase Assay

*Escherichia coli*/pET-28a and *E. coli*/pET28a-*ybhF* were cultured in LB liquid medium with 50 μg/ml kanamycin. The cells were cultured to an OD_600_ of approximately 0.6 at 37°C, and protein expression was induced with 1 mM IPTG for 4 h. The solubilized YbhF protein with His_6_-tag was purified. The ATPase activity of purified YbhF protein was measured. Assay of ATPase activity was conducted according to the method of by Balakrishnan et al. (2004) and Venter et al. (2008). Using the malachite green experiment, Pi released from ATP hydrolysis was measured. Purified YbhF protein (5 mg) was added to 100 mM HEPES buffer (pH 7.4) containing 2.5 mM MgSO_4_ and Na-ATP with various concentrations (0, 1.25, 2.5, 5.0, 10.0), and 30 μl of the above mixture was added to a 96-well plate and incubated at 30°C for 3 min and then 150 μl of freshly activated malachite green buffer (precooled before use) was added to stop the reaction. Incubation continued in the 96-well plate at 20°C for 5 min. Finally, 5 μl of 34% (w/v) citric acid was added to each well, and the cells were cultured in the dark for 30 min to allow color development. The OD_600_ of the samples was then measured in a spectrofluorometer (Infinite M200 PRO; Tecan, San Jose, CA, United States).

### EB Efflux Assay

The EB efflux assay was performed on *E. coli*/pET-28a, *E. coli*/pET28a-*ybhF*, *E. coli*Δ*ybhF*, *E. coliΔacrB*, *E. coli*Δ*acrB*Δ*ybhF*, *E. coli* ΔybhF/pET28a-*ybhF*, and *E. coli*Δ*acrB*/pET28a-*acrB* strains. The cells were cultured to an OD_600_ of approximately 0.6 at 37°C, and protein expression was induced with 1 mM IPTG for 4 h. The cell suspensions were washed three times with LB medium. When the OD_600_ reached 0.2, 2.5 μM EB was added, the cell suspensions were shaken at 37°C for 1 h to consume ATP and cellular energy, and then EB was preloaded in the energy-poor cells. The cells were washed three times in 50 mM KPi buffer (pH 7.0) containing 5 mM MgSO_4_. The cell suspensions were then incubated for 5 min at 37°C, then 25 mM glucose was added, and the EB efflux experiment was initiated. The excitation wavelength was 500 nm (9 nm slit), and the emission wavelength was 580 nm (20 nm slit). The EB efflux test was performed according to the protocol as described by Reuter et al. (2003); Balakrishnan et al. (2004), and Abdel-Motaal et al. (2018).

### Drug Resistance Assays

The micro-broth dilution method was used, and different concentrations of the antibacterial drug solution after dilution were separately added to a sterilized 96-well plate. The 1st to 11th wells were treated with a drug solution at 100 μl per well, and in the 12th well, the cells were added to serve as the growth control. Bacterial suspension concentration was corrected to 0.5 MacFarland units with Mueller Hinton (MH) broth, and after 1:1000 dilution with MH broth, 100 μl was added to each well. The cells were cultured approximately 16–20 h at 37°C, and the cell densities were determined using a spectrofluorometer (Infinite M200 PRO; Tecan, San Jose, CA, United States). The lowest drug concentration that completely inhibited the growth of bacteria in the wells was the minimum inhibitory concentration (MIC). The protocol of the MIC assay was according to Morita et al. (1998) and Ohene-Agyei et al. (2012). Three replicate wells were analyzed for each test, and the test was repeated at least three times.

### Tetracycline Efflux and Accumulation Assays

Tetracyclines have a conjugated system composed of benzene ring, ketone group, and enol; have two chromophiles, A ring and BCD ring; and have strong ultraviolet absorption and fluorescence properties in the near-visible light region. Therefore, we used a spectrofluorometer to analyze the efflux and accumulation of tetracycline (Yue et al., 2006). WT *E. coli*, *E. coli*Δ*ybhF*, *E. coli*Δ*acrB*, and *E. coli*Δ*acrB*Δ*ybhF* were cultured to an OD_600_ of 0.8 in LB medium. The cells were resuspended with Mg^2+^ buffer (pH 8.0) containing 50% methanol, 10 mM Tris–HCl (pH 8.0), 0.1 mM MgCl_2_, and 0.2% glucose (Sudano Roccaro et al., 2004). The cells were then washed with the same buffer two times. At the beginning of the reaction, tetracycline (100 μg/ml) was added to initiate the efflux assays. The intracellular tetracycline is immediately exported into the buffer, and the fluorescence of the tetracycline could be detected by a spectrofluorometer (Infinite M200 PRO; Tecan). The excitation wavelength was 400 nm (9 mm slit), and emission wavelength was of 520 nm (20 mm slit) (de Cristobal et al., 2006).

Because knockout of the efflux gene caused more drug to remain in the cell, the accumulated level of tetracycline was studied by using the same strains as the efflux experiments. After the OD_600_ reached 0.8, the cells were collected by centrifugation and resuspended with Mg^2+^ buffer, and 100 μg/ml of tetracycline was added. After incubation for 15 min, the bacterial suspension was centrifuged, and then resuspended in Mg^2+^ buffer. The absolute accumulated amounts of tetracycline in cells were calculated with a spectrofluorometer (Infinite M200 PRO; Tecan), with excitation and emission wavelengths of 400 and 520 nm, respectively (de Cristobal et al., 2006).

Another method was also used to detect the absolute accumulated amount of tetracycline in cells. The strains were cultured in LB liquid medium to an OD_600_ of 0.8. Then, 1-ml samples were suspended and washed with 100 mM Tris–HCl (pH 8.0) buffer and suspended again in 1 ml of 10 mM Tris–HCl (pH 8.0) buffer. Subsequently, 100 μg/ml tetracycline was added and incubated for 15 min. The cells were resuspended in 5 M HCl (1 ml). Samples were boiled at 100°C for 10 min, and tetracycline was quantitatively converted into anhydrotetracycline. The samples were centrifuged at 12,000 × *g* for 10 min, the supernatant was collected for subsequent experiments, and the anhydrotetracycline was measured by absorbance at 440 nm (Ball et al., 1980; de Cristobal et al., 2006).

### Inhibition of Tetracycline Transport Activity by *Ortho*-Vanadate, CCCP and MDR Substrates

Four strains were used in this assay, WT *E. coli*, *E. coli*Δ*acrB*, *E. coli*Δ*ybhF*, and *E. coli*Δ*acrB*Δ*ybhF*. *Ortho*-vanadate is a known ABC protein inhibitor that blocks hydrolysis of ATP and inhibits ATPase activity. CCCP is an uncoupler of the proton-motive force (Viveiros et al., 2008). To study whether tetracycline transport might be inhibited by *ortho*-vanadate (or CCCP), first we examined the effects of inhibitors in efflux experiments.

Cells were grown to an OD_600_ of 0.8 in LB liquid medium, to which 100 μg/ml tetracycline and *ortho*-vanadate at different concentrations (0, 0.5, 1, 1.5, 2, and 2.5 mM) or CCCP (0, 20, 40, 80, and 160 μM) were added. The detection of tetracycline efflux has been previously described (Li et al., 2014).

To determine whether the efflux of tetracycline could be inhibited by MDR substrates, we mixed five different concentrations of tetracycline (25, 50, 100, 150, and 200 μg/ml) with fixed concentrations of EB (or other MDR substrates) in Mg^2+^ buffer containing cells. Overall, four different concentrations of EB (2.5, 5, 10, and 20 μM) were examined. This method has been used to study the inhibitory kinetics of tetracycline against different drugs (Li et al., 2014). The tetracycline transport rate obtained with 200 μg/ml tetracycline and 0 μM EB (or another drug) was 1.0. The relative rates of each efflux curve were then calculated.

Tetracycline and different concentrations of EB or other inhibitory substrates were added to the cell suspensions, the efflux of tetracycline was detected by a spectrofluorometer, and the efflux rate of tetracycline was determined from the slope within the initial linear range. The efflux rate was specified as 1.0 without the inhibitor. Using Origin 8.0 software, a scatter plot was drawn first, changed it to log10 form, and then fitted to a non-linear curve using a four-parameter curve-fitting algorithm. The IC_50_ value was determined based on the concentration of the drug that had about 50% inhibitory effect on 200 μg/ml tetracycline efflux. The assays were performed according to the protocol as described by Li et al. (2014). The data were plotted as single substrate/single inhibitor kinetics using Origin 8.0 software.

### Growth Tests for NaCl Tolerance of *E. coli*△*ybhF*

Because overexpression of YbhF restored the salt sensitivity of KNabc and increased its resistance, we wondered whether the *ybhF* knockout strain, *E. coli*△*ybhF*, might be more sensitive to NaCl. The growths of *E. coli*/pET-28a, *E. coli*/pET28a-*ybhF*, and *E. coli*Δ*ybhF* in 0–0.6 M NaCl and under alkaline pH were tested in broth at 37°C for 12 h, and then the OD_600_ nm was measured every 1 h. The 12-h growth curve was then plotted using Origin8.0 software.

### Localization of YbhF by Immunoelectron Microscopy

Recombinant *E. coli*/pET-28a, *E. coli*/pET28a-*ybhF*, and *E. coli*Δ*ybhF* cells were cultured in LB liquid medium until the OD_600_ reached 0.6 at 37°C. Then, 1 mM IPTG was added and incubated for 4 h to induce protein expression. The cell suspensions were centrifuged at 4°C at 8000 × *g* for 10 min and then washed with phosphate-buffered saline (PBS) three times. The cells were then fixed overnight with 2.5% glutaraldehyde at 4°C. Using conventional electron microscopy preparation methods, the samples were embedded in Epon 812, followed by preparation of ultrathin sections of 70 nm thickness. The sections were treated with 1% H_2_O_2_ for 10 min, washed with PBS three times for 10 min each, and subsequently incubated with 1% bovine serum albumin for 30 min. The sections were then incubated overnight at 4°C with monoclonal antibody against YbhF protein (diluted 1:100) and then treated at room temperature for 1 h to allow the antibody to fully penetrate and bind. Goat anti-rabbit secondary antibody conjugated with 10 nm gold-label (diluted 1:100) was added and incubated at room temperature for 30 min. After washing with double-distilled water, the sections were stained with uranyl acetate and lead citrate for 1 min each. The sections were then examined using an electron microscope (JEOL, Tokyo, Japan). Immunoelectron microscopy studies were conducted according to the protocol of Freudl et al. (1986) and Kaufmann et al. (1994).

### Statistical Analysis

Each experiment was repeated at least three times. Student’s *t-*tests were used to analyze the differences among groups. Statistical significance was as follows: ^∗^*p* < 0.05; ^∗∗^*p* < 0.01; and ^∗∗∗^*p* < 0.001. Data are expressed as the mean ± standard deviation.

## Results

### Bioinformatics Analysis of the YbhFSR Transporter

**FIGURE 1:**
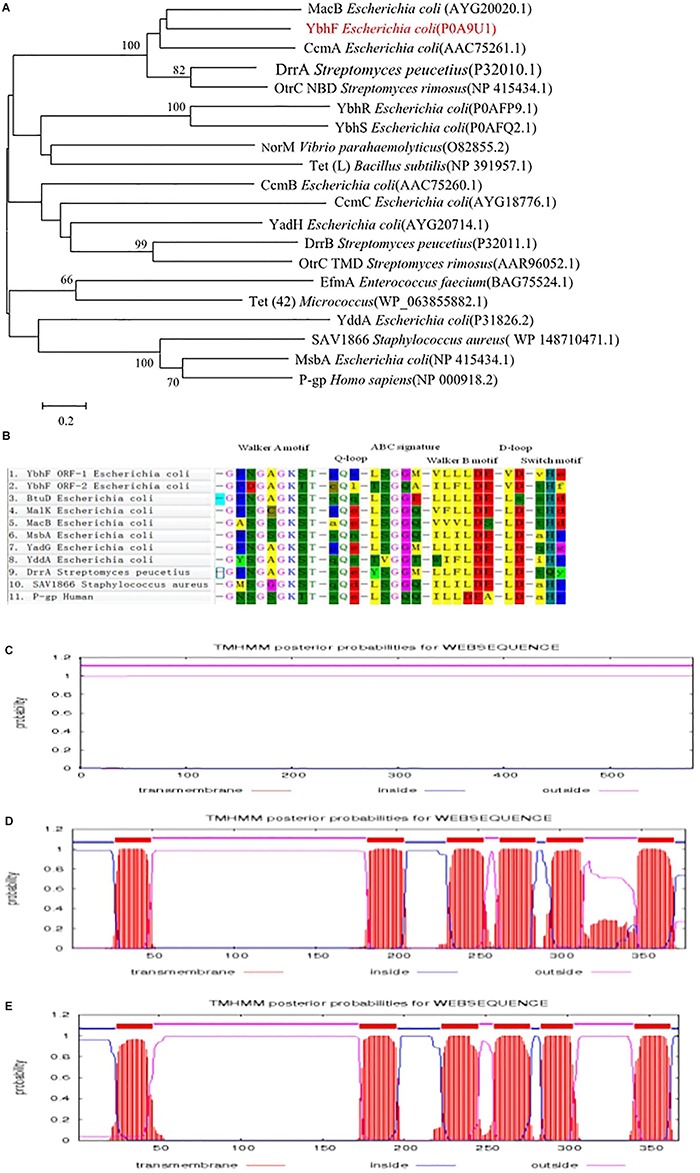
Bioinformatics analysis of the YbhFSR transporter. **(A)** Phylogenetic tree of YbhF. MEGA7.0 software was used to construct the phylogenetic tree of YbhF. **(B)** Alignment of the amino acid sequence of the YbhF-ORF1 region and YbhF-ORF2 region with other ABC drug-resistance transporters. There are at least six highly conserved motifs, in the order of walker A, Q-loop, ABC signature motif, walker B, D-loop and switch motif. TMHMM 2.0 software was used to predict transmembrane domain of YbhF **(C)**, YbhS **(D)**, and YbhR **(E)**. YbhF protein encoded no transmembrane helical domain. YbhS and YbhR have six putative α-helical transmembrane segments.

MEGA7.0 software was used to construct the phylogenetic tree of YbhF, which showed that YbhF with the macrolide drug efflux transporter MacB from *E. coli* and heme efflux transporter ccmA from *E. coli* first clustered together, followed by the doxorubicin and daunorubicin efflux transporter DrrA from *Streptomyces peucetiu*s (Kaur and Russell, 1998) and the oxytetracycline efflux transporter OtrC from *Streptomyces rimosus* (Yu et al., 2012) in a large group. MDR transporters of the ABC families, P-gp, MsbA, and Sav1866, clustered together. We speculated that if the YbhFSR transporter is a drug efflux transporter, it was probably similar to MacB or OtrC. MacB and OtrC are single-drug efflux pumps that transport only one drug or a class of closely related substrates (Figure 1A). ATP-binding subunits typically contain conserved sequence motifs that play important functional roles. YbhF contains two putative ATP-binding domains. An NCBI BLAST search for the amino acid sequence of the YbhF protein revealed many similar proteins, most of which were single-drug or multidrug resistance ABC transporters. Using DNAstar software, the conserved domain analysis of YbhF and other proteins showed that YbhF contained the conserved domains of Walker A, Q-loop, and Walker B, and signature motifs of D-loop and Switch motif (Figure 1B). These motifs are closely associated with ATP binding and hydrolysis, and conserved domain analysis indicated that YbhF is an ATP-binding cassette belonging to the ABC superfamily. Protparam software was used to predict the physicochemical properties and components of the amino acid sequence encoded by YbhF. The results showed that the *ybhF* gene encodes a protein of 578 amino acids, with a molecular weight of 63.76 kDa, a theoretical isoelectric point of 5.44, and with a slightly alkaline pH. TMHMM 2.0 software predicted that the YbhF protein encoded no transmembrane helical domain, YbhS and YbhR have six putative α-helical transmembrane segments and belong to the TMDs (Figures 1C–E), suggesting that YbhFSR form ({TMD}_2_-{NBD}_2_) structural units. Using EMBL software, blast amino acid sequence 334–574 of the YbhF protein with RB6469, a sodium ABC transporter ATP-binding protein from *Rhodopirellula baltica* SH 1 (Gene ID: 1794229). The result shows that the amino acid sequence identity of these two proteins was 31.3% and the similarity was 55.2% (Supplementary Figure S3).

### Expression and Purification of YbhF

**FIGURE 2:**
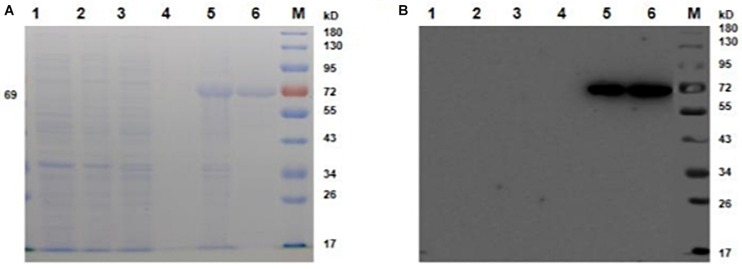
Expression and purification of YbhF. *E. coli* BL21 cells were transformed with the plasmid pET28a, and the expression of YbhF was induced by IPTG. **(A)** SDS-PAGE was used to analyze the expression and purification of YbhF. **(B)** Western blot was used to analyze the expression and purification of YbhF. Primary antibody: Anti-His antibody, secondary antibody: HRP-labeled goat anti-mouse IgG. M: Prestained Protein Ladder, (1) Before induction of *E. coli*/pET28a. (2) After induction of *E. coli*/pET28a. (3) Before induction of *E. coli*/pET28a-*ybhF* whole cells. (4) Supernatant after induction of *E. coli*/pET28a-*ybhF* whole cells. (5) Precipitation after induction of *E. coli*/pET28a-*ybhF* whole cells. (6) Purified YbhF protein.

*Escherichia coli* cells were transformed with the plasmid pET28a, and the expression of YbhF was induced by IPTG. SDS-PAGE electrophoresis with Coomassie Brilliant Blue staining was used to analyze the expression of YbhF (Figure 2A). The results showed that the target band appeared at 69 kDa after induction, indicating that the recombinant YbhF protein existed in the precipitation as inclusion body. The recombinant YbhF protein was purified by a nickel column, and the protein purity was high. Western blot was performed using Anti-YbhF antibody as the primary antibody and detect the expression of YbhF (Figure 2B). Molecular weight of the band was the same as predicted.

### The ATPase Activity of YbhF

**FIGURE 3:**
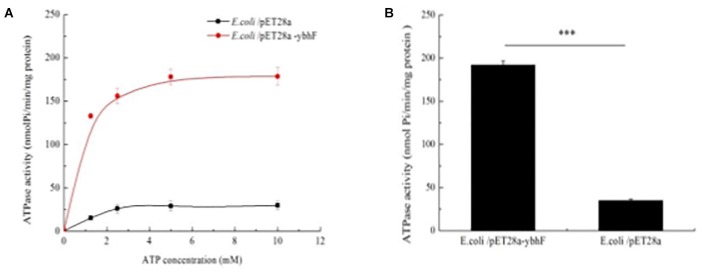
ATPase assay of overexpressing-YbhF and WT-YbhF protein. **(A)** The ATPase activity of YbhF with different ATP concentrations. ATPase activity was detected through the Malachite Green method. ATPase activity of purified YbhF proteins was determined from the liberation of Pi from ATP. **(B)** ATPase activity. The WT-YbhF protein was 35.1 ± 3.4 nmol Pi/min/mg protein, overexpressing-YbhF protein was 191.8 ± 3.9 nmol/min/mg protein. ^∗^*p* < 0.05; ^∗∗^*p* < 0.01; and ^∗∗∗^*p* < 0.001. Data are expressed as the mean ± standard deviation.

The ATPase activity of purified overexpression and WT-YbhF proteins was detected with different ATP concentrations using the malachite green method (Figure 3A). The results showed the ATPase activity of overexpression-YbhF with a maximum activity (*V*_max_) of 191.8 ± 3.9 nmol/min/mg, but that of the WT YbhF was only 35.1 ± 3.4 nmol/min/mg (Figure 3B). Affinity for ATP of overexpression-YbhF (*K*_m_ = 0.5 ± 0.1) was significantly higher than WT (*K*_m_ = 1.2 ± 0.1) (Table 2). These results were consistent with those from bioinformatics analysis, indicating that the YbhF protein had ATPase activity and that YbhFSR transport of substrates utilized the energy released by ATP hydrolysis.

**TABLE 2:** ATPase activities of purified YbhF proteins.

	*K*_m_ (mM)	*V*_max_ (nmol/min/mg)
Overexpression-YbhF	0.5 ± 0.1	191.8 ± 3.9
WT -YbhF	1.2 ± 0.1	35.1 ± 3.4

### EB Efflux of YbhFSR

**FIGURE 4:**
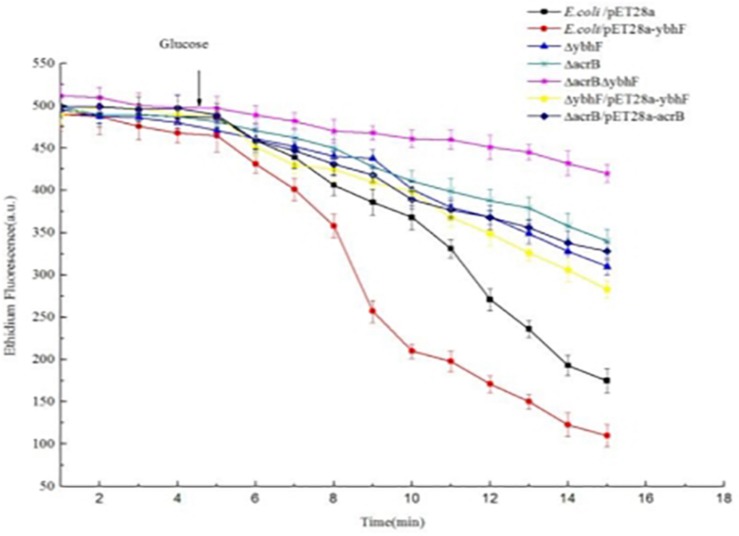
Efflux assay of ethidium bromide (EB) by whole cells of *E. coli* and its transformants. Energy-starved cells were pre-loaded with EB at a final concentration of 2.5 μM. After 5 min (downward arrow), glucose was added to cell suspensions at a final concentration of 25 mM to re-energize cells. The efflux of intracellular EB was monitored continuously by measuring the fluorescence of EB at the excitation and emission wavelengths of 500 and 580 nm, respectively.

The EB fluorescent probe was used to detect whether YbhFSR mediated efflux transport using hydrolysis of ATP. Figure 4 shows that the EB efflux efficiency of YbhF-expressing *E. coli*/pET28a-*ybhF* was higher than that of the empty vector containing *E. coli*/pET28a cells. The abilities of *E. coli*△*acrB*, *E. coli*△*ybhF*, and *E. coli*△*acrB*△*ybhF* to perform EB efflux were significantly lower, and the efflux ability of the *E. coli*△*acrB*△*ybhF* double knockout was the lowest, but the complement strains can restore the function of the knockout strains, indicating that the knockout of genes did not produce polarity and the *ybhF* gene knockout reduced the ethidium efflux capacity.

### Resistance of YbhFSR to Drugs

The MICs of different drugs were detected in *E. coli*/pET28a, *E. coli*/pET28a-*ybhF*, *E. coli*△*acrB*, *E. coli*△*ybhF*, *E. coli*△*ybhS*, *E. coli*△*ybhR* and *E. coli*△*acrB*△*ybhF*, *E. coli*/pET28a, and *E. coli*△*acrB* as the controls. The results showed that when the *ybhF* gene and *ybhR* gene were knocked out respectively, the bacteria showed increased sensitivity to tetracycline drugs such as tetracycline, oxytetracycline, chlortetracycline, doxycycline, and two other cationic substrates (EB and Hoechst33342), but not to other multidrug efflux substrates such as rifampicin, norfloxacin, ofloxacin, and doxorubicin. Table 3 shows that when compared with the *E. coli*△*acrB* sensitive strain, the *E. coli*△*acrB*△*ybhF* double-knockout strain showed a significantly lower MIC value, as well as a significantly lower efflux ability for the four tetracyclines. The MIC of tetracycline *E. coli*△*acrB*△*ybhF* was only 0.125 μg/ml, a value significantly lower than those of *E. coli*/pET28a and *E. coli*△*acrB*. We therefore speculated that the YbhFSR transporter was a single-drug efflux transporter of tetracyclines.

**TABLE 3:** Minimal inhibition concentrations (MICs) of the tested antimicrobial drugs.

Drug	Minimum inhibitory concentrations (μg/ml)						
	*E.coli*/pET28a	*E.coli/*pET28a-*ybhF*	*E.coli*△*ybhF*	*E.coli*△*ybhS*	*E.coli*△*ybhR*	*E.coli* △*acrB*	*E.coli*△*acrB△ybhF*
Tetracycline	1	2	0.5	1	0.5	0.25	0.125
Oxytetracycline	32	128	16	32	16	8	4
Chlortetracycline	64	64	1	64	1	0.125	< 0.125
Doxycycline	32	32	1	32	1	0.125	< 0.125
Ethidium	256	256	128	256	128	128	16
Hoechst	128	128	64	128	64	64	32
Cefoperazone	1	1	1	1	1	0.125	0.125
Cefazolin	64	64	32	64	64	32	32
Streptomycin	> 256	>256	> 256	>256	> 256	>256	> 256
Ampicillin	8	8	8	8	8	4	4
Roxithromycin	> 256	>256	> 256	>256	> 256	>256	> 256
Chloramphenicol	32	32	32	32	32	0.5	0.5
Rifampicin	16	16	16	16	16	8	8
Norfloxacin	2	2	2	2	2	< 0.125	<0.125
Deoxycholate	> 256	>256	> 256	>256	> 256	>256	> 256
Sodium cholate	> 256	>256	> 256	>256	> 256	>256	> 256
Ofloxacin	0.125	0.125	0.125	0.125	0.125	< 0.125	<0.125
Doxorubicin	4	4	4	4	4	4	1
Daunorubicin	64	64	64	64	64	1	1
Acridine flavin	0.5	0.5	0.5	0.5	0.5	0.5	0.5
Quinine	> 256	>256	> 256	>256	> 256	0.5	0.5

### Efflux and Accumulation of Tetracycline

**FIGURE 5:**
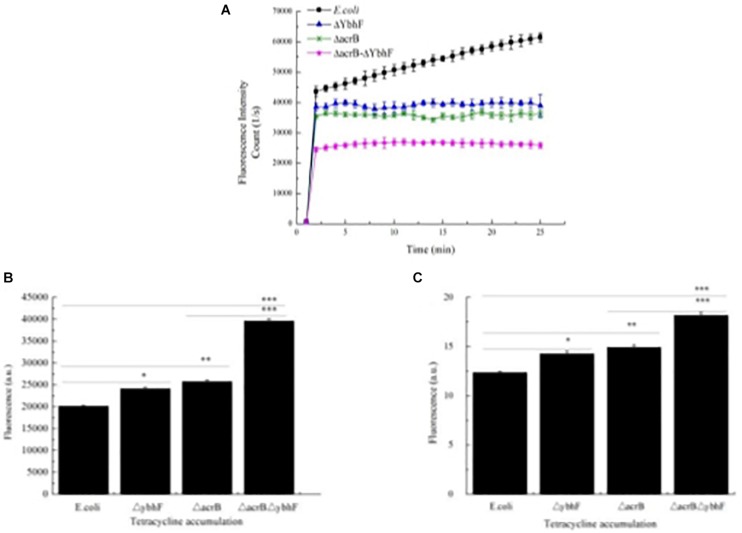
Tetracycline efflux and accumulation assays. **(A)** Tetracycline efflux. Bacteria were loaded with tetracycline and efflux measured using spectrofluorometry, there was a slower efflux from the *E. coli*Δ*acrB*, *E. coli*Δ*ybhF* than in the *E. coli* cells, the outflow speed is the slowest in *E. coli*Δ*acrB*Δ*ybhF* strains. **(B)** Tetracycline accumulation. Bacteria were loaded with tetracycline, pelleted, and resuspended in Mg^2+^ buffer, in which released fluorescence (direct measurement of accumulated tetracycline) was immediately detected with a spectrofluorometer. The fluorescence was detected at excitation of 400 nm (9-mm slit), and emission was monitored at 520 nm (20-mm slit). **(C)** Total accumulation of tetracycline determined after conversion into anhydrotetracycline. The anhydrotetracycline was measured by absorbance at 440 nm. Tetracycline efflux and accumulation assays were repeated three times. ^∗^*p* < 0.05; ^∗∗^*p* < 0.01; and ^∗∗∗^*p* < 0.001. Data are expressed as the mean ± standard deviation.

Increased sensitivity to tetracycline may be due to decreased drug efflux. To verify this possibility, we used the autofluorescence characteristics of tetracycline measured with a spectrofluorometer (Infinite M200 PRO; Tecan) to detect the efflux and accumulation kinetics of tetracycline in different sensitive strains. The results showed that the outflow speed was slowest in *E. coli*△*acrB*△*ybhF*, and there was a slower efflux in *E. coli*△*acrB* and *E. coli*△*ybhF*, when compared with the WT *E. coli* (Figure 5A). Both *ybhF* and *acrB* knockouts reduced tetracycline efflux, while double knockouts further reduced tetracycline efflux.

Because impaired tetracycline efflux would result in greater drug retention in cells, we next studied the levels of intracellular accumulation of tetracycline in the same strains used in the efflux assay. The cells were treated with tetracycline (100 μg/ml) for 15 min at 37°C. The bacterial suspensions were then centrifuged and resuspended in Mg^2+^ buffer, allowing the tetracycline to be released from cells into the buffer, which was detected with a fluorescence spectrometer to quantitatively determine the amount of tetracycline accumulated during the 15-min loading phase. Figure 5B shows that the *E. coli*△*acrB*△*ybhF* mutation resulted in significantly higher intracellular tetracycline levels, and the accumulations of tetracycline in the cells of *E. coli*△*acrB* and *E. coli*△*ybhF* were higher than that of the WT *E. coli* control strain, and WT *E. coli* had the lowest accumulation of tetracycline.

Another method to detect the accumulation of tetracycline in cells involved measurement of the conversion of tetracycline to anhydrotetracycline. Figure 5C shows that *E. coli*△*acrB*△*ybhF* accumulated higher levels of tetracycline than *E. coli*△*acrB* and *E. coli*△*ybhF*, and *E. coli*Δ*acrB* and *E. coli*△*ybhF* accumulated higher levels than WT *E. coli*.

Overall, the results of the efflux and accumulation of tetracycline showed that knockout of *ybhF* decreased the efflux of tetracycline and increased the intracellular accumulation.

### Inhibition of Tetracycline Transport Activity by *Ortho*-Vanadate, CCCP and MDR Substrates

**FIGURE 6:**
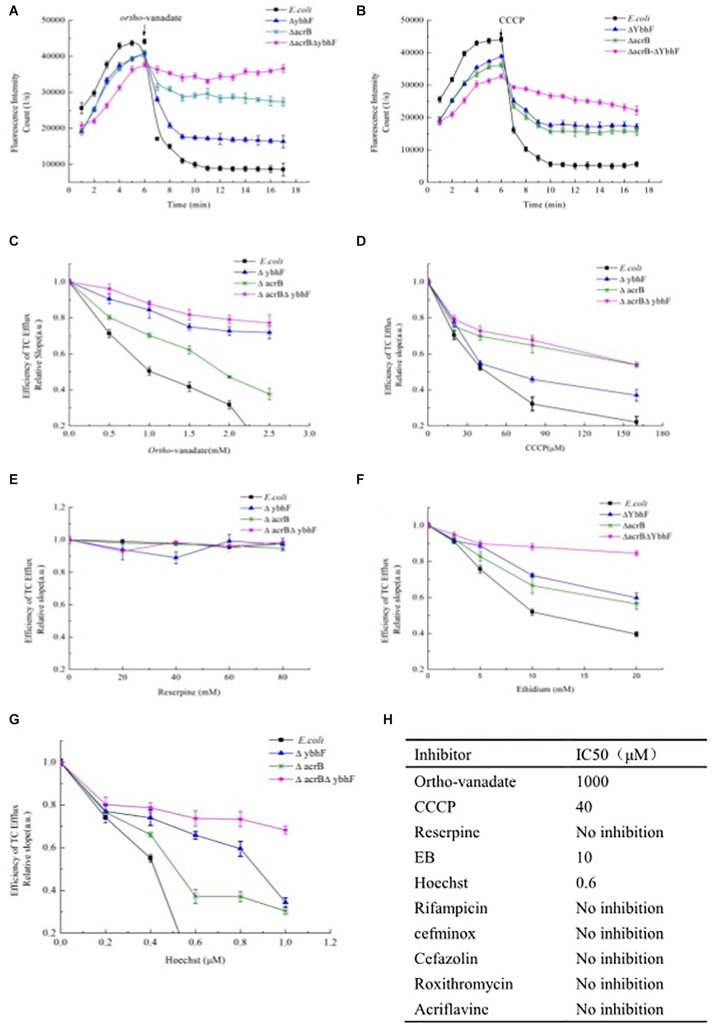
Inhibition of tetracycline (TC) transport activity by *ortho*-vanadate, CCCP, and MDR substrates. **(A)** *ortho*-vanadate (1 mM) inhibited efflux of tetracycline (TC). **(B)** CCCP (40 μM) inhibited efflux of tetracycline (TC). CCCP had a stronger inhibitory effect on tetracycline efflux than *ortho*-vanadate. **(C)** Kinetic analysis of the inhibitory effect of *ortho*-vanadate on tetracycline (TC) efflux activity. The assay was carried out in the presence of increasing concentrations of *ortho*-vanadate ranging from 0 to 2.5 mM. **(D)** Kinetic analysis of the inhibitory effect of CCCP on tetracycline (TC) efflux activity. The experimental conditions were the same as for panel C. The assay was carried out in the presence of increasing concentrations of CCCP ranging from 0 to 160 μM. **(E)** Kinetic analysis of the inhibitory effect of reserpine on tetracycline (TC) efflux activity. The assay was carried out in the presence of increasing concentrations of reserpine ranging from 0 to 80 μM. **(F)** Kinetic analysis of the inhibitory effect of EB on tetracycline (TC) efflux activity. The assay was carried out in the presence of increasing concentrations of EB ranging from 0 to 20 mM. **(G)** Kinetic analysis of the inhibitory effect of Hoechst33342 on tetracycline (TC) efflux activity. The assay was carried out in the presence of increasing concentrations of Hoechst33342 ranging from 0 to 1.0 μM. **(H)** A table showing a summary of the IC 50 values. The IC 50 values were calculated as described under *Materials and methods*.

In whole cells, while maintaining the concentration of tetracycline (100 μg/ml), when the concentration of *ortho*-vanadate (0.5–2 mM) or CCCP (20–160 μM) increased gradually, we found that *ortho*-vanadate (Figure 6A) and CCCP (Figure 6B) both inhibited the efflux of tetracycline after *ybhF* was knocked out, with an IC_50_ of 1.0 mM and 40 μM, respectively. CCCP had a stronger inhibitory effect on tetracycline efflux than *ortho*-vanadate. Subsequently, we added tetracycline at 100 μg/ml to different cultures for 5 min and then tested the inhibition of tetracycline efflux by adding 1.0 mM *ortho*-vanadate or 40 μm CCCP, which showed a highly significant decrease in fluorescence. The most obvious decrease after the addition of *ortho*-vanadate was in *E. coli*, followed by *E. coli*△*acrB*, whereas the results in *E. coli*△*ybhF* were similar to *E. coli*△*acrB*△*ybhF*, with no significant decrease in fluorescence (Figure 6C). After addition of CCCP, the four strains all showed a decrease in fluorescence. The most obvious decrease in fluorescence was in *E. coli*, followed by *E. coli*△*ybhF*. The fluorescence values of *E. coli*△*acrB* and *E. coli*△*acrB*△*ybhF* also decreased, but the magnitude was smaller than that in the other two strains (Figure 6D). We also used another MDR inhibitor, reserpine, but it had no effect on the efflux and accumulation of tetracycline (Figure 6E).

To determine whether the YbhFSR transporter recognized and interacted with other known multidrug substrates, we investigated the inhibitory effects of different drugs on tetracycline efflux in whole cells. In this assay, while maintaining the concentration of tetracycline (100 μg/ml), different concentrations of multidrug efflux substrates were added, as described in the experimental methods. The results showed that only two substrates, EB (Figure 6F) and Hoechst33342 (Figure 6G), inhibited excretion of tetracycline with high efficiency, whereas the other drugs such as rifampicin, cefminox, cefazolin, roxithromycin, and acriflavine showed no inhibition (Figure 6H). These results indicated that the YbhFSR transporter recognized tetracycline and two known cation substrates, EB and Hoechst33342, which could be transferred by almost all drug efflux transporters, but it did not recognize other MDR substrates, indicating that the transporter YbhFSR was a drug efflux transporter that recognized and transferred tetracycline, EB, and Hoechst33342.

### Resistance of YbhFSR to Salts and Alkaline pH

**FIGURE 7:**
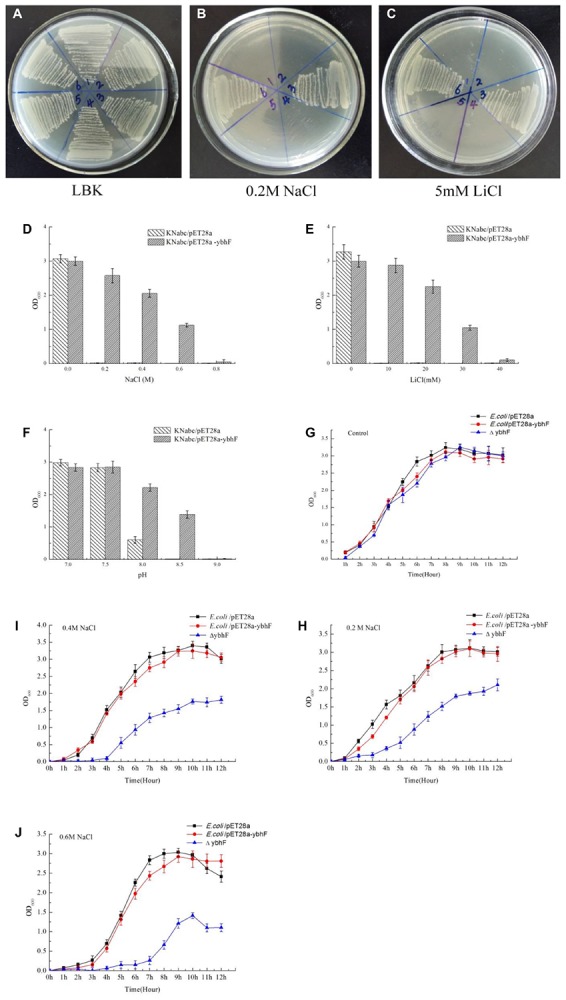
Growth tests for *E. coli* KNabc, KNabc/pET28a-*ybhF*, and KNabc/pET28a under saline or alkaline stress (**A–C)**. For the complementation test, *E. coli* KNabc transformant cells were grown on the LBK medium plates, 0.2 M NaCl or 5 mM LiCl. 1, 4. KNabc, 2, 5. KNabc/pET28a, 3, 6. KNabc/pET28a-*ybhF*. KNabc/pET28a and KNabc/pET28a-*ybhF* were grown in the LBK media containing 0-0.4 M NaCl **(D)** or 0–20 mM LiCl **(E)** or with the addition of 50 mM NaCl at the pH values from 7.0 to 9.0 **(F).** Cell growth was ended after 24 h and detected the OD_600_ nm. Growth tests for NaCl tolerance of *E. coli*/pET28a, *E. coli*/pET28a-*ybhF*, and *E. coli*Δ*ybhF*. Cells were grown in LB liquid medium, take each time point of liquid measuring OD_600_ nm, and draw the growth curve of these strains. **(G)** Control, growth of cells in LBK medium. **(H)** 0.2 M NaCl. **(I)** 0.4 NaCl. **(J)** 0.6 M NaCl. Compared with the *E. coli* and *E. coli*/pET28a-*ybhF*, *E. coli*△*ybhF* growth was significantly reduced in 0.4 and 0.6 M NaCl.

The growth of *E. coli* KNabc, *E. coli* KNabc/pET28a-*ybhF*, and *E. coli* KNabc/pET28a under different conditions of NaCl and LiCl were examined. The results showed that *E. coli* KNabc and *E. coli* KNabc/pET28a grew in LBK, but not in 0.2 M NaCl or 5 mM LiCl. *E. coli* KNabc/pET28a-*ybhF* showed normal growth in LBK, 0.2 M NaCl and 5 mM LiCl (Figures 7A–C). Subsequently, *E. coli* KNabc/pET28a and *E. coli* KNabc/pET28a-*ybhF* were used in a more detailed growth test involving the salt resistance and alkaline pH values. As the concentration of NaCl, LiCl, and the pH increased, *E. coli* KNabc/pET28a-*ybhF* grew in 0.6 M NaCl or 15 mM LiCl, but *E. coli* KNabc/pET28a did not grow under the same stress conditions (Figures 7D,E). Likewise, *E. coli* KNabc/pET28a-*ybhF* was resistant to an alkaline pH of 8.5 in the presence of 50 mM NaCl, but *E. coli* KNabc/pET28a did not grow at all under the same stress conditions (Figure 7F). Thus, we speculated that YbhFSR was an Na^+^ (Li^+^)/H^+^ antiporter.

Because YbhFSR can transport Na^+^, we then determined the growth curves under different Na^+^ concentrations after *ybhF* knockout. The results were consistent with our expectations; compared with the *E. coli* control strain, *E. coli*△*ybhF* only grew well in 0.2 M NaCl (Figures 7G,H), whereas the growth was significantly reduced in 0.4 and 0.6 M NaCl (Figures 7I,J). However, the control *E. coli* strain and the overexpressing *E. coli*/pET28a-*ybhF* strain grew normally under this Na^+^ stress. Growth of the recombinant strains (△*ybhF*, △*ybhS*, △*ybhR*) were also determined under alkaline pH values (Supplementary Figure S4). At pH 9.5, △*ybhF* and △*ybhR* grew significantly higher than control strains (Supplementary Figure S4E). Together, the results indicated that YbhFSR had a Na^+^ (Li^+^) transport function and was a Na^+^ (Li^+^)/H^+^ antiporter.

### Localization of YbhF Using Immunoelectron Microscopy

**FIGURE 8:**
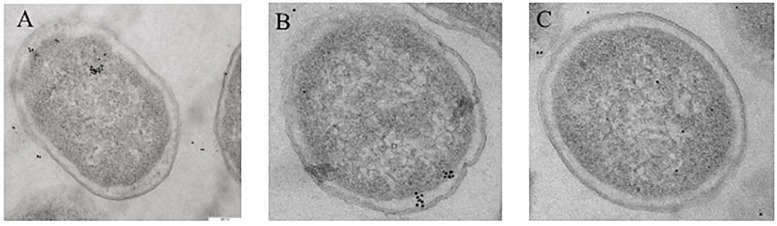
The subcellular localization of YbhF in *E. coli*/pET28a, *E. coli*/pET28a-*ybhF*, and *E. coli* Δ *ybhF*. Whole cells were subjected to immunogold labeling with antibody against YbhF. **(A)** *E. coli*/pET28a; only a small amount of scattered distribution was in the vicinity of the cell inner membrane. **(B)** *E. coli*/pET28a-*ybhF*; the YbhF protein is clustered in the vicinity of the cell inner membrane. **(C)** *E. coli* Δ*ybhF*; only few colloidal gold particles in the cytoplasm and no one in the vicinity of the cell inner membrane.

After super-bore sectioning of recombinant *E. coli*/pET-28a, *E. coli*/pET28a-*ybhF*, and *E. coli*Δ*ybhF*, monoclonal antibodies against YbhF were used as the primary antibody, and colloidal gold-labeled goat anti-rabbit IgG was used as a secondary antibody for immunostaining. Transmission electron microscopy (JEM-2100Plus) indicated that the *E. coli*/pET-28a empty plasmid strain had very few immunopositive regions for YbhF protein, and a small scattered distribution was observed in the vicinity of the cell inner membrane, possibly due to the presence of endogenous YbhF protein in cells. In the *E. coli*/pET28a-*ybhF* overexpression strain, the positive signal was obvious, showing that the YbhF protein clustered in the vicinity of the inner cell membrane. In the *E. coli*Δ*ybhF* knockout strain, almost no colloidal gold signal was detected near the cell membrane, whereas a few colloidal gold particles were present in the cytoplasm (Figure 8).

## Discussion

The *E. coli* K-12 strain is a non-virulent and deficient O-antigen and K-antigen strain widely used in the laboratory (Riley et al., 2006; Moussatova et al., 2008). It is often considered to be the standard strain of *E. coli*. In 1997, the genome of the K-12 *E. coli* strain was sequenced (Blattner et al., 1997). The largest protein family in the *E. coli* K-12 strain is the ATP-binding cassette family, accounting for 5% of the entire genome. From these genomic data, all encoded ABC proteins were analyzed, of which seven were possible drug export transporters, and two of them, MsbA (Singh et al., 2016) and MacB (Kobayashi et al., 2001), were confirmed as drug export transporters. MsbA is a multidrug efflux pump (Reyes et al., 2006) and lipid A transporter (Siarheyeva and Sharom, 2009), and MacB is a single-drug efflux pump, which can transfer macrolide drugs (Tikhonova et al., 2007), whereas the functions of the other five have only been partially reported. YbhFSR is a putative ABC-type drug efflux transporter in *E. coli*, located in the ybiHYbhGFSR operon, which comprises five genes: *ybiH*, *ybhG*, *ybhF*, *ybhS*, and *ybhR.* Analysis of the amino acid sequence indicated that the *ybiH* gene belonged to the TetR family of transcriptional regulators. The first confirmed TetR family member in *E. coli* is Tn10 (Kaneko et al., 1985; Morsczeck et al., 2004), which controls the expression of the tetracycline efflux pump gene. The second gene is *ybhG*, encoding a predicted MFP family member. The resistance–nodulation–division (RND) and some ABC exporters usually transport drugs outside the cells, together with outer membrane channel proteins and MFP. In this operon, the *ybhG* gene belonging to the MFP family member. The MFP connects the inner membrane pump (YhbFSR) to the outer membrane channel (probably TolC). For example, in the RND pumps, e.g., AcrAB-TolC, the MFP is AcrA. There are three functional genes in this operon: YbhF possesses the NBDs, and YbhS and YbhR are the TMDs; they all comprise six hypothetical α-helical transmembrane segments. In many ABC transporters, the transmembrane motif is fused to the NBD, forming a single TMD-NBD structural unit. The production of functional dimers is generally assumed to be represented as {TMD-NBD}_2_. These transporters typically include multidrug-resistant P-gp from mammals (Teodori et al., 2006) and its homologs, MsbA and LmrA, from bacteria (van den Berg Van Saparoea et al., 2005; Venter et al., 2008; Singh et al., 2016). In P-gp, two NBDs and two TMDs are fused to one polypeptide, while in MsbA and LmrA, one TMD fuses with one NBD to form a semi-transporter, and the semi-transporter homodimerizes to form a full transporter. However, in some of the bacterial ABC transporters, the NBD and the TMD are encoded by different proteins, forming ({TMD}_2_-{NBD}_2_) structural units (Young and Holland, 1999). ABC drug transporters comprise SDR transporters and MDR transporters. SDR transporters are specifically used to transport one or a group of closely related drugs. Almost all SDR transporters have the same structural unit of {TMD}_2_-{NBD}_2_ (Ross et al., 1990), but MDR transporters often exist as {TMD-NBD}_2_, such as P-gp (Prochazkova et al., 2012), MsbA (Chang, 2003), and LmrA (van Veen et al., 1998). However, there are exceptions. For example, MacAB exporter has the structural unit of ({TMD}_2_-{NBD}_2_), but its function was a single-drug efflux pump. The YbhFSR system consists of three proteins, with YbhF having two NBDs, and YbhR and YbhS each containing a TMD domain. The YbhFSR transporter has a {TMD}_2_-{NBD}_2_ arrangement. Therefore, according to the classification of domain arrangements of ABC drug efflux transporters in single-drug efflux and multidrug efflux, YbhFSR belongs to the single-drug efflux pump group. We constructed a phylogenetic tree between YbhF and other ABC efflux pumps and found that YbhF first clustered with the macrolide efflux transporters, MacB and ccmA, followed by two other single-drug efflux transporters, DrrA and OtrC, aggregated into a large group. The other multidrug efflux transporters, P-gp, MsbA, and Sav1866, are grouped together. Based on these considerations, both domain arrangement and cluster analysis supported YbhFSR as a single-drug efflux transporter. Subsequently, we knocked out and overexpressed the *ybhF* gene of the ATP-binding domain in the YbhFSR transporter, which destroyed the integrity of the YbhFSR transporter and affected its function (Orelle et al., 2019). The MIC assay confirmed YbhFSR efflux tetracyclines, EB, and Hoechst33342. In the drug sensitivity experiments, the *E. coli*/pET28a-*ybhF* overexpression strain did not show an increase in resistance to all tetracyclines. This may be related to the overexpression of YbhF alone rather than to the co-expression with YbhR and YbhS. However, the knockout of *ybhF* resulted in a significant decrease in the MIC values of tetracyclines, EB, and Hoechst33342. At the same time, there was inhibited efflux and an increased intracellular accumulation of tetracycline and EB. In the efflux experiments involving tetracycline, three inhibitors (*ortho*-vanadate, CCCP, and reserpine) were used. The results showed that *ortho*-vanadate significantly inhibited efflux, indicating that it inhibited YbhFGSR, which was consistent with the previous analysis of YbhF and the ATPase activity test results. The inhibitory effect of CCCP was significantly higher than that of *ortho*-vanadate, and the fluorescence value was greater. CCCP is an uncoupler of the proton-motive force that significantly inhibits tetracycline efflux of the AcrAB system, and the resistance of AcrAB to tetracycline has been previously confirmed (Nolivos et al., 2019). The *E. coli*Δ*acrB*Δ*ybhF* double knockout strain also showed significant fluorescence reduction after the addition of CCCP. Taken together, these results indicated that, in addition to YbhFSR and AcrAB, there are other efflux pumps using proton gradients, such as TetA (Kaneko et al., 1985), involved in the efflux of tetracycline. However, we cannot exclude the possibility that YbhFSR itself might also be involved in the proton-coupled efflux of substrates. We also found that the YbhFSR exporter can transfer Na^+^ and Li^+^. At least two ABC transporters have been reported that can use proton-motive force to transporter substrates. For example, MsbA from *E. coli* (Singh et al., 2016) and LmrA from *L. lactis* (Venter et al., 2003; van den Berg Van Saparoea et al., 2005; Velamakanni et al., 2009) reported multidrug efflux transporters belonging to the ABC family that also used a proton pump to facilitate transmembrane transport of substrates. YbhFSR transporters are similar to LmrA that they are both drug exporters and can also transport Na^+^ (Velamakanni et al., 2009). Based on the above results, we speculated that the YbhFSR transporter is a drug efflux transporter that depends on ATP hydrolysis to provide energy and can also use proton-motive force to transporter substrates. In our experiments, the AcrB system played an important role in tetracycline efflux. However, another efflux system, YbhFSR, also played an important role.

Although there is convincing evidence that bacterial drug transporters have protective functions, there are many reports confirming that these transporters actually have specific physiological functions (Schinkel, 1997; Johnstone et al., 2000; Teodori et al., 2006; Poole, 2008). Bacterial cells can simultaneously encode multiple drug transporters. For example, the genome of *E. coli* encodes at least 29 drug efflux transporters (Nishino and Yamaguchi, 2001). There is increasing evidence that there are redundant drug efflux pumps in bacteria and that these efflux pumps have their own physiological functions under normal conditions (Sarkadi et al., 2006; Mizutani et al., 2008). Regarding the physiological function of YbhFSR, domain analysis showed a NatA domain between amino acids 300 and 580 in YbhF, which was involved in the transport of Na^+^. Using the *E. coli* KNabc, we found that pET28a-*ybhF* restored the ability of *E. coli* KNabc to transport Na^+^ after overexpression in this strain. Subsequently, the salt tolerance experiments also confirmed that YbhFSR transported Na^+^ and Li^+^.

YbhFSR is one of the uncharacterized ABC transporters in *E. coli*. Little was previously known regarding the function of YbhFSR. In this study, we showed that YbhFSR, an ABC transporter, exported tetracycline, EB, Hoechst and may also be a Na^+^(Li^+^)/H^+^ antiporter. The YbhFGSR transporter consists of YbhF (NBD), YbhS and YbhR (TMD), and YhbG, the MFP. When this transporter effluxes substrates, it must function as a complete system. This paper focuses on the NBD domain YbhF, while the two TMDs, YbhS and YbhR, were less studied. The transporter with the highest similarity to YbhFSR in *E. coli* is the lipoprotein transporter LolCDE. LolC and LolE are TMDs and LolD is the NBD. Both LolC and LolE are essential for the release of lipoproteins. Based on the current results, it is still uncertain whether the YbhS and YbhR are both essential for the transport of tetracyclines. While it is possible that one of them plays a primary role and the other plays a secondary role, it is more likely that both subunits are required for efflux activity. Subsequent in-depth research on the role of YhbS and YhbR in tetracycline efflux will be of great reference value for explaining the function of YbhFSR transporter and for studying the evolution of ABC multidrug transporter. In addition, several export systems of Gram-negative bacteria require additional components to function properly, such as a MFP and an outer membrane protein. TolC is a major outer membrane channel in *E. coli* and it plays an important role in the excretion of various molecules across the outer membrane (Horiyama et al., 2010; Krishnamoorthy et al., 2013). TolC functions in combination with three types of transport systems: ABC-type transporters, RND-type transporters, and Major Facilitator superfamily (MFS) (Koronakis et al., 2004). The trans-envelope complex consists of transporters that span the entire envelope to export the substrates directly into the external medium (Krishnamoorthy et al., 2013). The AcrAB (RND-type transporters) and MacAB efflux pumps (ABC-type) in *E. coli* share the same outer membrane channel TolC to export their substrates (Lu and Zgurskaya, 2012). The MFP family protein YbhG is encoded in the same operon that encodes YbhFSR. Therefore, it is likely that YhbG performs the sane functions as other MFPs in *E. coli* and connects the YhbFSR pump to an outer membrane channel protein, such as TolC. This is supported by the results of several recent studies indicating that TolC is an important component of the YbhFSR pump (Yamanaka et al., 2016; Babu et al., 2017; Mateus et al., 2018). Therefore, the likely subunit composition of the functional form of this efflux pump is YhbFGSR-TolC.

ATP-binding cassette proteins comprise the largest family in *E. coli* proteins. Although *E. coli* is a model organism, with many of its gene functions well-known, there are still many genes that have not been characterized. Functional studies of the *ybhF* gene provide theoretical support for further understanding of the ABC family. The YbhFSR transporter is encoded by three different genes encoding the two TMDs and two NBDs. This transporter is therefore a good model to study how the different domains of the ABC drug transporter interact to complete ATP hydrolysis and release energy, as well as how energy is transferred to the TMD and how transfer of substrates is completed. Overall, the organizational structure of YbhFSR makes it a good model for studying the evolution of ABC exporters.

## Data Availability Statement

The raw data supporting the conclusions of this article will be made available by the authors, without undue reservation, to any qualified researcher.

## Author Contributions

ZYF and YC designed the experiments. ZYF, DL, and YW conducted the experiments. ZYF and ZL analyzed the experimental results. ZZ, JC, and ZWF analyzed the sequencing data. LW provided monoclonal antibody of YbhF protein. LG and SY used Origin8.0 software to plot the experimental results. ZYF and BS wrote the manuscript. ZYF, DL, and YC reviewed and modified the manuscript.

## Conflict of Interest

The authors declare that the research was conducted in the absence of any commercial or financial relationships that could be construed as a potential conflict of interest.

## Footnotes

1 https://evomics.org/resources/software/bioinformatics-software/clustal-x/

2 https://www.megasoftware.net/

3 https://www.dnastar.com/

4 https://web.expasy.org/protparam/

5 http://www.cbs.dtu.dk/services/TMHMM/

6 https://www.ebi.ac.uk/Tools/psa/emboss_needle/
