# Supplementary material for: A Putative Efflux Transporter of the ABC Family, YbhFSR, in Escherichia coli Functions in Tetracycline Efflux and Na+(Li+)/H+ Transport
Source: Front Microbiol. 2020 Apr 23;11:556. doi: 10.3389/fmicb.2020.00556 (PMC7190983; doi:10.3389/fmicb.2020.00556)
Supplement: Supplementary file 1 [file Data_Sheet_1.pdf]

Supplementary Material

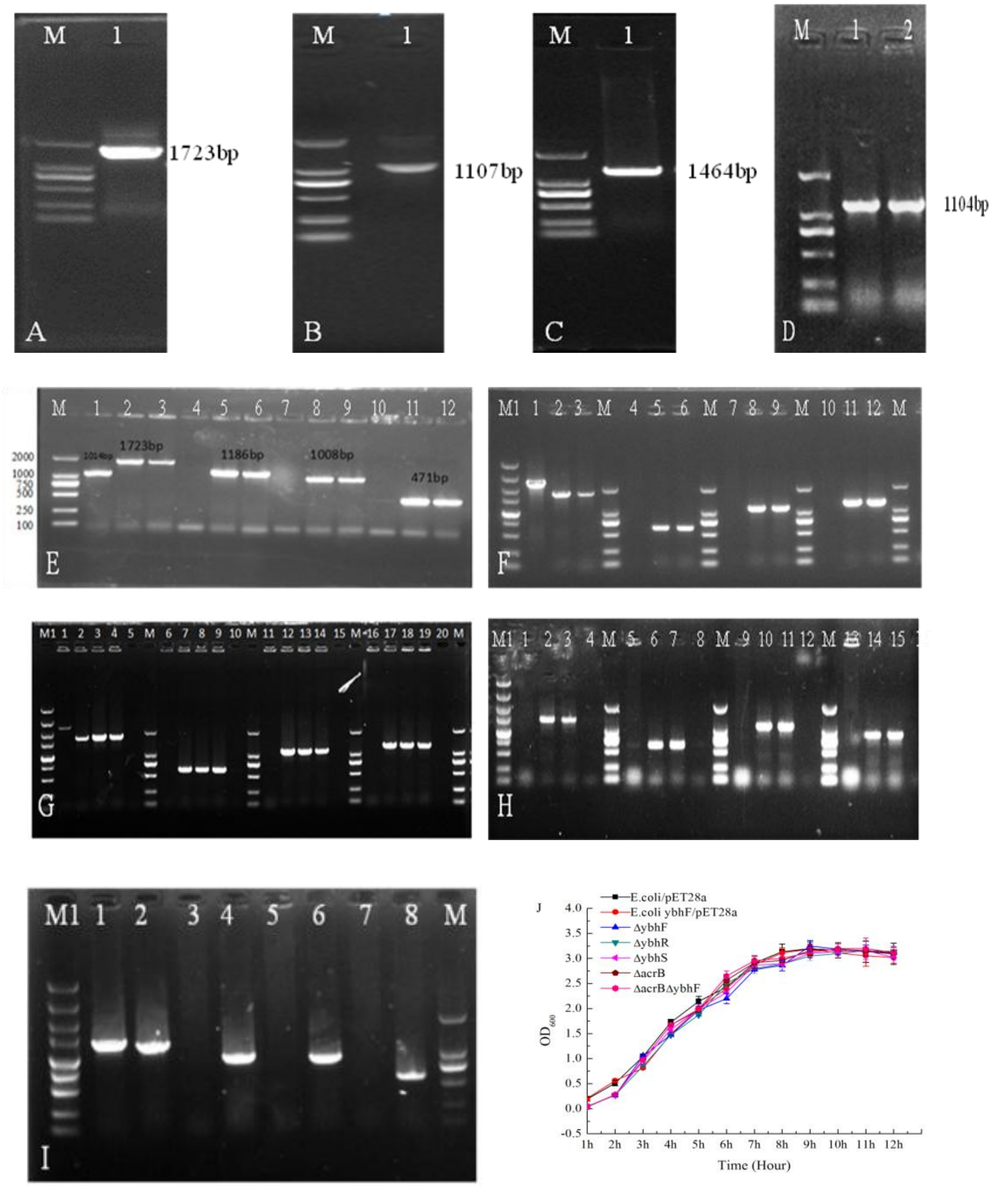

Fig 1 | Knockout and verification. M1. DL5000; M. DL2000 **(A)** PCR amplification results of recombinant DNA fragmen. M. DL2000 Marker, 1. Target fragment of *acrB* (1723bp). **(B)** Target fragment of *ybhF* (1107bp). **(C)** PCR verification of the pKD46 plasmid was transformed into *E. coli* K-12. 1. pKD46 (1464bp). **(D)** Target fragment of *ybhS* (2) *and ybhR* (3) (1104bp).**(E)** Results of positive clones by PCR of Δ*acrB*. **(F)** Results of positive clones by PCR of Δ*ybhF*. **(G)** Results of positive clones by PCR of Δ*acrB*Δ *ybhF*.**(H)** Results of positive clones by PCR of Δ*ybhR*. **(I)** Results of positive clones by PCR of Δ*ybhS*. **(J)** Growth curve of the recombinant strains. Take each time point of liquid measuring OD_{600} nm, draw the growth curve of these strains.

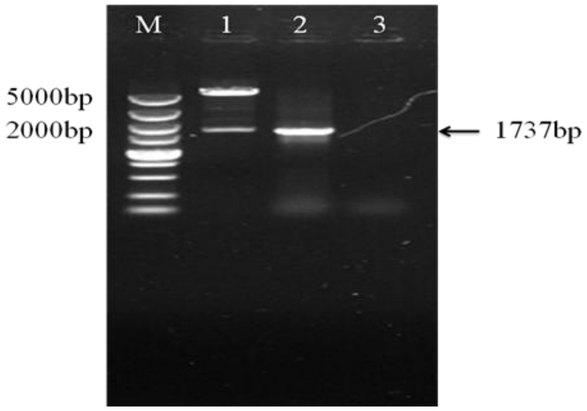

Fig 2 │ Identification of recombinant *E. coli* /pET28a-*ybhF* plasmid by PCR analysis and restriction enzyme digestion. M. DL5000. 1. Double enzyme digestion verification of the *ybhF* gene. 2. PCR amplification verification. 3. negative control.

NP_415315.4	------LTKKF----GDFAATDHVNFAVKRGEIFGLLGPNGAGKSTTFKMMCGLLVPTSG
NP_867276.1	MLLIDSLTKRFSIESGTVHAVDGLSMRVAPGEVFGLLGPNGAGKTTTLRMVLGLLEPDDG
NP_415315.4	QALVLGMDLKESSGKARQHLGYMAQKFSLYGNLTVEQNLRFFSGVYGLRGRAQNEKISRM
NP_867276.1	FAEVAGIRTSKDPFAAKAKLGFVSASDGVYPWLSVREMLLYFADLYGVAPQQATARLKEL
NP_415315.4	SEAFGLKSIASHATDELPLGFKQRLALACSLMHEPDILFLDEPTSGVDPLTRREFWLHIN
NP_867276.1	ASVMQIEALLDRRAGSLSTGQRQRVTLVRGLIHDPPVMLLDEPTRGLDVVGVQTIFEYIE
NP_415315.4	SMVEKGVTVMVTTHFMDEAE-YCDRIGLVYRGKLIASGTPDDLKAQSANDEQPDPTMEQA
NP_867276.1	HLRAAGKAVVVCTHRLDEAERLCDQFGLLHRGRIRYRGTLNDLREETGREH-----LVEM
NP_415315.4	FIQLIHDWD----KEHSNE
NP_867276.1	FVDLMNSTDPALTEDHA--

Fig 3 Sequence alignment of the YbhF protein (334–574) was blasted with RB6469, a sodium ABC transporter ATP-binding protein from *Rhodopirellula baltica* SH 1 (Gene ID: 1794229). NP_415315.4 was YbhF. NP_867276.1 was RB6469. The amino acid sequence identity of these two proteins was 31.3%, and the similarity was 55.2%.

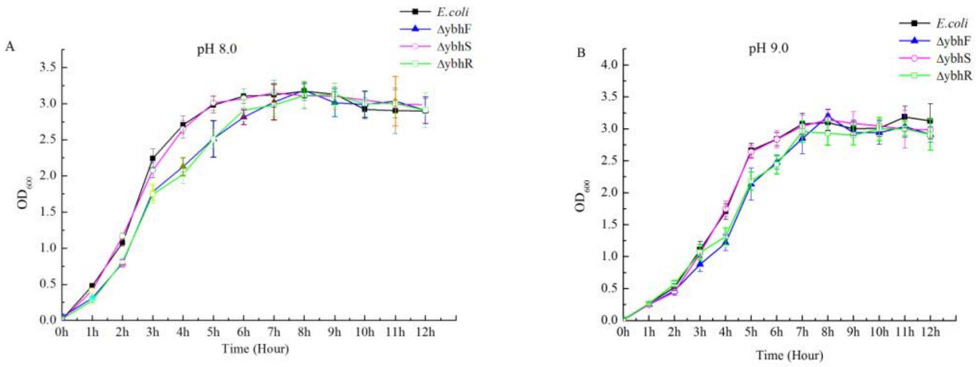

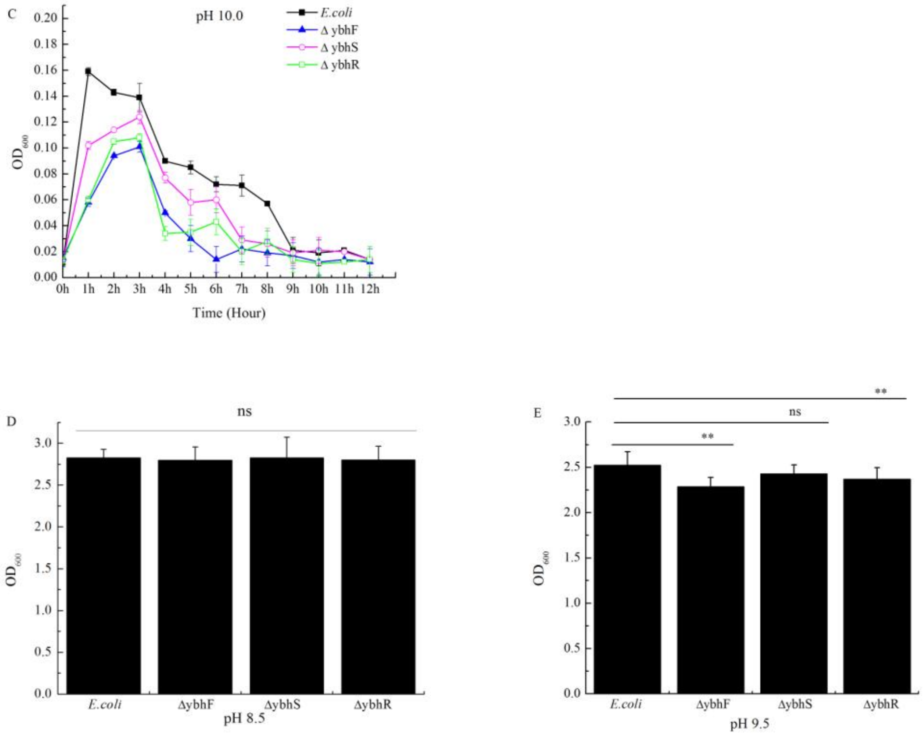

Fig 4| Growth curve of the recombinant under alkaline pH. Take each time point of LB medium measuring OD value of 600 nm, draw the growth curve of *E. coli*, *E. coli* Δ*ybhF*, *E. coli* Δ*ybhS E. coli* ΔybhR cells at the pH values 8.0 **(A)** pH values 9.0 **(B)** pH values 10.0 **(C)**. Cell growth was ended after 24 h and monitored turbidimetrically of 600 nm at the pH values 8.5 **(D)** and pH values 9.5 **(E)**.

Table 1 |Primers used in this experiment

primer	Sequence(5'to3')	Size(bp)
ybhF-F	CGCGGATCCATGAATGATGCCGTTATCACG	30
ybhF-R	CCCAAGCTTTTACTCATTGCTATGCTCCTTATCC	34
pKD46 F	AGAGCTGGGCGCGTCACTAC	20
pKD46 R	TCGTACTGTTTTCCCCAGGC	20
K-*acrB* F	GGATACCGCTGCGGCAATCCGTGCTGAACTGGCGAAGATGGAACCGTGTAGGCTGGAGCTGCTTC	71
K-*acrB* R	CAACGTTGTTCTTTTCTTTGGTCAGATAGTAATGCGTTACCTCATCATATGAATATCCTCCTTAG	66
K-*ybhF* F	CCAGGGAATGCCAGTGACGGTACAATTCGGTGACGAGGCAGGACTTGAGCGATTGTGTAGGCTGGAG	68
K-*ybhF* R	CCAGGGAATGCCAGTGACGGTACAATTCGGTGACGAGGCAGGACTTGAGCGATTGTGTAGGCTGGAG	68

K-*ybhS* F	AAGCCTTTATTCAGTTGATCCACGACTGGGATAAGGAGCAT AGCATTGAGCGATTGTGTAGGCTGGAG	68
K-*ybhS* R	TCTTTGCGGATTAACGTCCATAAGCGATGAAACATGCTCTTCTC CTAACGGCTGACATGGGAATTAGC	68
K-*ybhR* F	CGACGTGGCTGAAAACCAAACGTCGGCTGGATTAGGGAGAAGA GCTTGAGCGATTGTGTAGGCTGGAG	68
K-*ybhR* R	CTTGCCGGCCCGGCGGTAGTGTCTTTGCTGGCGAAAGATAAGA AGTAACGGCTGACATGGGAATTAGC	68
T-*acrB* F	GAACTACGACATCATCGCAGAG	22
T-*acrB* R	TCAAGGAAACGAACGCAATAC	21
T-*ybhF* F	GTGCTGCTTTATACCGATGG	20
T-*ybhF* R	TAAGCGGTTCAAAAGTCTGC	20
T-*ybhS* F	ATGGAGCAAGCCTTTATTCA	20
T-*ybhS* R	CGCCGTTATCTTCATCGTAG	20
T-*ybhR* F	GTTCAGCTGATTGAAGATGCGG	22
T-*ybhR* R	GATGTTGTTGGTCCACTGGTA	21
Cm F	TGAAACTCACCCAGGGATTG	20
Cm R	ATAAATCCTGGTGTCCCTGT	20
Kan F	CAGTCATAGCCGAATAGCCT	20
Kan R	CGGCCACAGTCGATGAATCC	20
